# Dynamics of nonspherical, fractal-like water-ice particles in a plasma environment

**DOI:** 10.1038/s41598-018-33854-5

**Published:** 2018-10-18

**Authors:** Kil-Byoung Chai

**Affiliations:** Korea Atomic Energy Research Institute, Daejeon, 34057 South Korea

## Abstract

Plasmas containing small solid-state particles (also known as dust particles) are ubiquitous in nature and laboratories. Existing models typically assume that the dust particles are spherical but several observations and simulations indicate that a significant amount of dust particles are nonspherical. Because dust particles are not spherical they show different dynamics from spherical particles in a plasma environment namely, they align in the direction perpendicular to the force equilibrium line, rotate about their alignment axis due to the interaction between the dipole moment and the surrounding electric field, and show vortex motion while maintaining their alignment and rotation when they are exposed to a nonconservative drag force.

## Introduction

Dusty plasmas are ubiquitous in nature and laboratories. Notable examples are astrophysical molecular clouds^1^, protoplanetary disks^2^, certain of planetary rings^3,4^, terrestrial polar mesospheric clouds^5^, fusion devices^6^, and plasma processing^7^. In these plasmas, nonspherical dust particles are frequently observed; in interstellar mediums, molecular clouds, and protoplanetary disks, the linear polarization of starlight, which results from the elongated dust particles being aligned along the surrounding magnetic field, has been observed^8,9^. It is proposed that ice particles in polar mesospheric clouds are also elongated based on spectral measurements of the light scattered by the ice particles^10^. In fusion plasmas, dust particles, which are considered to be potential radioactive hazards due to their high chemical reactivity with tritium, are observed in various shapes including fluffy and flake geometries^11,12^.

Although a significant amount of dust particles observed in naturally occurring and laboratory plasmas are nonspherical, the dust particles are typically assumed to be spherical^2,3,5^ and the number density is assumed to have power-law dependence on the particle radius^2^. In addition, most diagnostics for particle size and density are based on the spherical assumption. Thus, the spherical assumption may result in some discrepancy between observations and analysis. We believe that the asphericity might be the key to solve some of open questions in related fields such as the radial-drift barrier that sub-meter-sized dust particles fall onto the central star owing to the drag of sub-Keplerian gas before they form a planetesimal in protoplanetary disks^13^ and abnormally strong radar reflections from the polar mesospheric clouds^14^. Several attempts have been made to address some of the unsolved problems using nonspherical dust shape^15,16^.

However, there are only limited numbers of studies on nonspherical dust particles because it is hard to make nonspherical particles in laboratories. Du *et al*. and Dap *et al*. reported that nonspherical, fluffy particles can be formed by the agglomeration of spherical particles in plasma environments^17,18^. Yousefi *et al*. studied the charging and rotation of nonspherical aggregates using a laboratory experiment and a simulation code^19^. Recently, the experiments developed at Caltech and KAERI have successfully created nonspherical dust particles in a plasma environment^20–22^ and hence they provide a good opportunity to study the dynamics of nonspherical particles. In this work, by using a laboratory experiment which can produce nonspherical water-ice particles in a plasma environment we report the dynamical properties of nonspherical, fractal-like water-ice particles. We believe that the results of the present study provide an intuition on how a nonspherical, fractal shape affects the force balance and dynamics of particles in space and laboratory dusty plasmas.

Besides the dynamics of nonspherical particles, our results are intriguing because dust particles are made of water-ice. In many astrophysical situations, water-ice is particularly important because i) water-ice is one of the most abundant solid materials in protoplanetary disks (including our solar system) and molecular clouds^1^, ii) water-ice plays an important role in star and planetesimal formations such as cooling the circumstellar gas^23^ and sticking other particles like a glue^24^, and iii) water has the key to understanding the origin of life.

## Experimental Setup and Sequence

**Figure 1:**
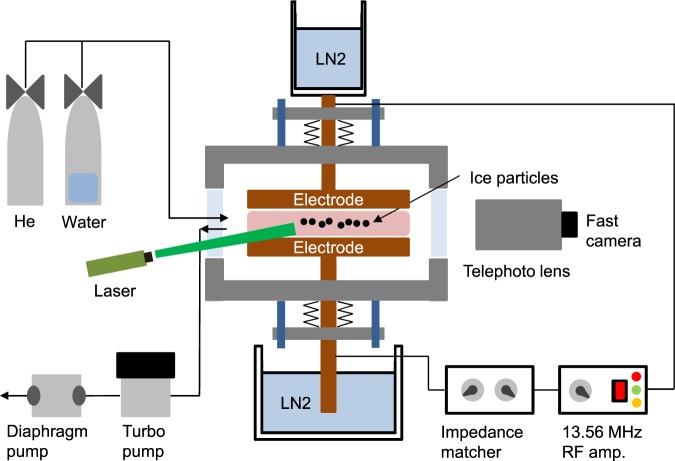
Sketch of our laboratory apparatus. Plasmas are obtained between two parallel electrodes made of copper and connected to liquid nitrogen cooling systems. A 13.56 MHz RF is used to ignite the plasma with helium gas. In order to trace ice particle motions, a green laser sheet is illuminated to the particles formed and levitating in a plasma and then scattered images from the particles are recorded by a CMOS camera at 300 fps. It is noticeable that the temperature of the upper electrode is always cooler than that of the bottom electrode probably due to the better thermal contact of the upper electrode cooling system. Thus, thermophoretic force acts on the ice particles upwards while the gravitational force is exerted downwards.

Figure 1 shows a sketch of our laboratory apparatus. Similar to that reported by Shimizu *et al*.^25^, the electrodes made of copper are connected to both a 13.56 MHz RF generator and liquid nitrogen cooling systems; the upper electrode is connected to a copper cup holding the liquid nitrogen while the lower electrode dips into the liquid nitrogen. The radius of the electrodes is 3 cm and the gap distance between the two electrodes is 1.5 cm. Plasma is ignited between the two electrodes using 1–2 W of RF power with helium gas in this work.

In order to study the dynamics of ice particles formed in our experiment, we shine a green laser sheet with a thickness of 1 mm and a height of 1 cm on ice particles. A fast framing camera that is able to capture movies at up to 575 fps (IDS, UI-3130-CP) is used to record the motion of the ice particles. The camera has a resolution of 800 × 600 pixels and each pixel corresponds to 8.6 μm.

The typical experimental sequence is that i) plasma is ignited with helium gas at a pressure of 600 mTorr after the two electrodes are cooled down by liquid nitrogen, ii) water vapor is directly introduced to the plasma, iii) a nucleation of ice particles takes place, and iv) the helium pressure is decreased to 400 mTorr and the inlet water vapor valve is closed to stop the further nucleation of ice particles. As the pressure decreases, water-ice particles grow quickly to hundreds μm in length. After the growth process ends, vortex flows start to appear in the bulk plasma region.

## Results and Discussion

**Figure 2:**
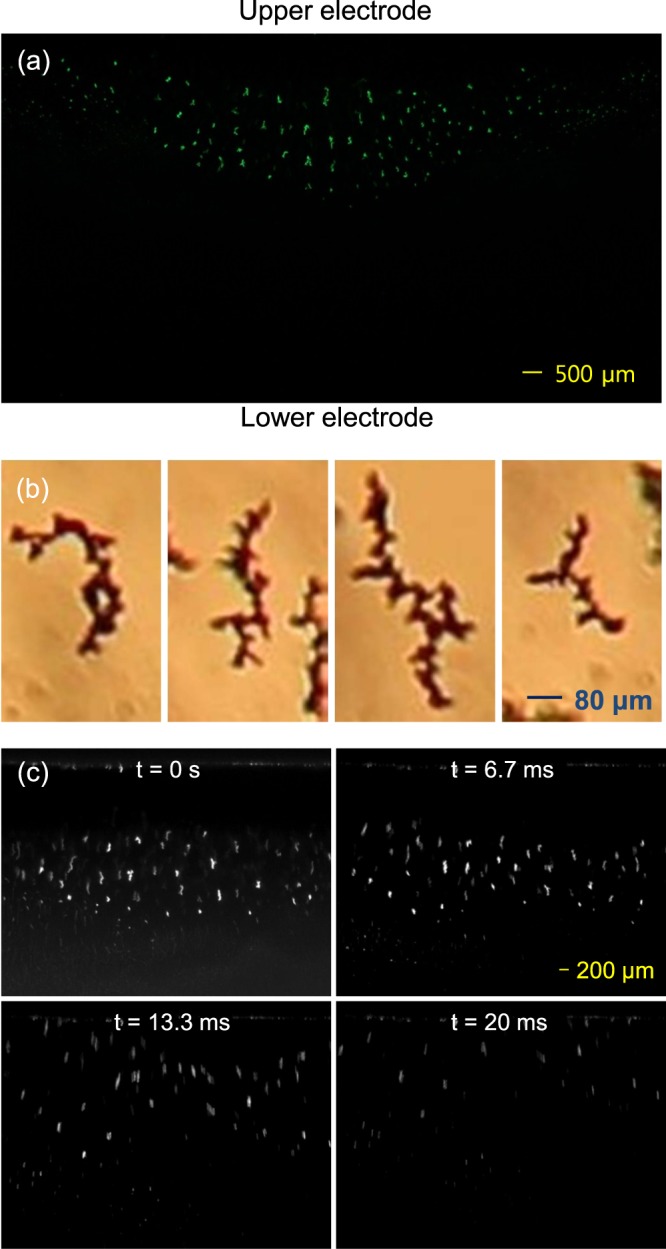
Alignment and force balance of nonspherical water-ice particles in a plasma. (**a**) Cloud of water-ice particles observed in the laboratory experiment and (**b**) high-resolution images of ice particles. Ice particles formed and levitating in our lab experiment are typically nonspherical (elongated) and have a fractal nature. In addition, the elongated particles typically align in a direction perpendicular to the force equilibrium line (or dust cloud edge). The observation that a dust cloud is observed only in the upper part of the plasma indicates that thermophoretic force is stronger than the gravitational force. (**c**) Motion of ice particles after the plasma is terminated. Most particles move upward after the plasma is turned off because the thermophoretic force overcomes the gravitational force.

Figure 2(a,b) show water-ice particles formed in our plasma experiment (the helium pressure was 300 mTorr in these cases). The images were captured by a Canon EOS 600D camera. As shown in the figures, most particles grow nonspherically. The high-resolution 2-D images shown in Fig. 2(b) reveal that the ice grains formed in helium plasmas have a fractal nature (see also ref.^22^) and the fractal dimension is 1.7 which is obtained using the box counting method embedded in ImageJ^22^. Another interesting feature is that the elongated particles line up in the direction perpendicular to the edge line of a dust cloud. The reason for the alignment will be discussed later.

Figure 2(a) also reveals that the dust cloud is only formed in the upper part of the plasma. This is due to the fact that the thermophoretic force exerted on the ice particles is larger than the gravitational force and the electrostatic force. The temperature of the upper electrode is always cooler than that of the bottom electrode^21^ because the thermal contact of the upper cooling system is better than that of the lower system. In the experiment, the equilibrium temperatures of the upper and bottom electrodes are 120 K and 140 K, respectively, measured by a resistance temperature detector (RTD). Thus, the thermophoretic force resulting from the temperature gradient of neutral gas is exerted on ice particles upward and is given by^26^

1 $${{\bf{F}}}_{{\boldsymbol{t}}{\boldsymbol{h}}}=-\frac{32}{15}\frac{{r}_{d}^{2}}{{V}_{th,n}}{\kappa }_{n}{\rm{\nabla }}{T}_{n}(1+\frac{5\pi }{32}(1-\alpha )).$$

Here, *r*_*d*_ is the radius of ice particles, *V*_*th*,*n*_ is the thermal speed of neutral gas, *κ*_*n*_ is the thermal coefficient (*κ*_*n*_ = 0.13 for helium at 200 K), *T*_*n*_ is the temperature of neutral gas, and *α* is the accommodation coefficient. Since ice particles are typically lined up along the gradient of the neutral gas temperature, we can use the minor radius (15 μm) as an effective radius for the thermophoretic force. Assuming ∇*T*_*n*_ = 20 K/1.5 cm and *α* = 1, the thermophoretic force is obtained to be 9.6 × 10^−11^ N.

The electrostatic force is expressed as **F**_**es**_ = – *eZ*_*d*_**E** where *Z*_*d*_ is the number of charges residing on a dust particle and **E** is the ambipolar electric field. Using the orbital motion limited theory with a spherical assumption, we obtain *Z*_*d*_ = (0.3–1.2) × 10^5^ (depending on 15 μm < *r* < 60 μm) and using the 2-D diffusion solution, *E*_*z*_ is obtained to be 100–1000 V/m (depending on the vertical position). Therefore, the electrostatic force is estimated as 1 × 10^–12^ N <** F**_**es**_ < 2 × 10^–11^ N.

The gravitational force is expressed as **F**_**g**_ = *m*_*d*_**g**, where *m*_*d*_ is the mass of the ice particles. Assuming ice particles as a cylinder with a radius of 15 μm and a length of 120 μm (nominal size of the ice particles formed in He 300–400 mTorr plasmas), the gravitational force is estimated to be *F*_*g*_ = 7.6 × 10^−10^ N. This simple calculation shows that the thermophoretic force exerted on the elongated particles is several times larger than the electrostatic force as we expected but is smaller than the gravitational force which is in conflict with our observation.

However, it is evident that the thermophoretic force is greater than the gravitational force. All the ice particles levitating between the two electrodes move upward (towards the upper electrode) when the plasma is turned off as shown in Fig. 2(b) (also see M1 which is an animated file). Thus, we can obtain an important intuition regarding on nonspherical, fractal-like particles: the mass estimated based on the shape is likely overestimated.

It was found that most of the elongated particles generated in our experiment line up along the vertical axis except for the particles levitating in the left and right sides of the dust cloud; the alignment orientation of these particles is slightly different from the vertical axis and is perpendicular to the edge line of the dust cloud. No horizontally aligned particles are observed from our experiment.

**Figure 3:**
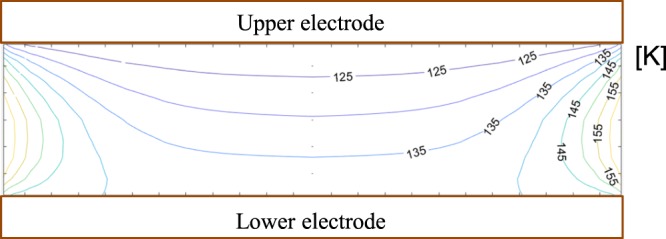
Calculated neutral gas temperature profile between the two electrodes. The neutral gas temperature is obtained by solving the time-independent heat equation (Laplace equation). Temperatures of the lower electrode, upper electrode, and chamber wall are assumed to be 140 K, 120 K, and 300 K, respectively. The contour plot of the temperature seems similar to the shape of the observed dust clouds.

It is generally accepted that elongated particles line up along or perpendicularly the electric field direction inside the plasma. The electric field induces dipole and quadrupole moments on the particles and either vertical or horizontal alignment is a stable equilibrium depending on the aspect ratio when the torque balance equation is solved^27^. However, this cannot explain our observation because particles levitating left and right sides of the dust cloud do not line up along the electric field direction. They rather line up along the gradient of the neutral gas temperature shown in Fig. 3. Figure 3 shows the isothermal lines between the two electrodes which were obtained by solving the time-independent heat equation with boundary conditions that the temperature of the bottom electrode is 140 K, the temperature of the upper electrode is 120 K, and the temperature of the wall is 300 K. Thus, it is reasonable to think that the thermophoretic force provides a restoring torque to align along its direction. An alternative explanation is that because ice particles are elongated, the charges residing on the particles are concentrated on both ends of the particles. Then, two neighborhood particles tend to align perpendicular to the force equilibrium line to minimize their mutual electric potential energy^22^.

**Figure 4:**
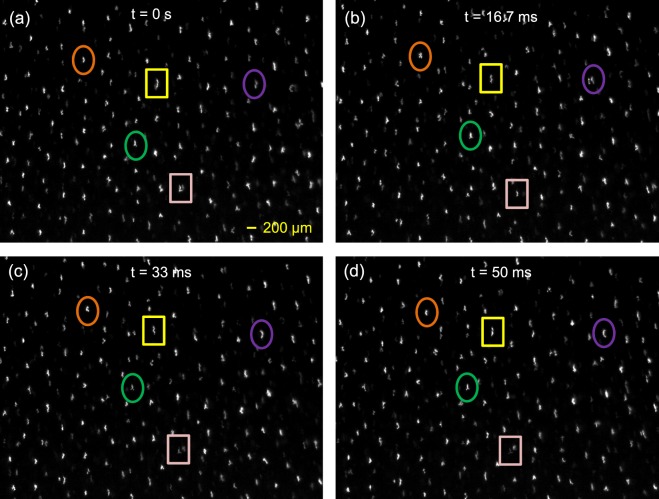
Rotation of nonspherical water-ice particles about their alignment axis in a plasma. (**a**–**d**) Sequential images showing that ice particles rotate about their alignment axis. The time step between images is 1/60 s. It is clearly seen that most particles rotate about their alignment axis and the rotation frequency ranges between 20 and 50 Hz. The rotation results from the interaction between the horizontal intrinsic dipole moment and the horizontal electric field inside plasma^19^.

The nonspherical ice particles rotate about their alignment axis as shown in Fig. 4(a–d). A multimedia file is also available online (M2). The fast framing camera recorded the central region where ice particles move slowly. The scale bar and time of each image are displayed in the figure. The rotational frequency ranges between 20 and 50 Hz and thus the angular speed of the rotation (*ω*) ranges between 125 and 625 rad/s. The cause of the rotation is due to the interaction between the horizontal component of the ice particle dipole moment and the electric field^19^. The governing equation for the rotational motion is expressed as^19^

2 $$\frac{d\overrightarrow{L}}{dt}=I\frac{d\overrightarrow{\omega }}{dt}={\overrightarrow{\tau }}_{E}+{\overrightarrow{\tau }}_{D}$$

where ${\overrightarrow{\tau }}_{E}={\boldsymbol{p}}\times {\boldsymbol{E}}$ is the torque due to the horizontal electric field and ${\overrightarrow{\tau }}_{D}={\boldsymbol{r}}\times {{\boldsymbol{F}}}_{{\boldsymbol{nd}}}$ is the torque due to the neutral drag force^28^ **F**_**nd**_ = −(4/3)π*r*_*d*_^2^*n*_*n*_*V*_*th*,*n*_*m*_*n*_***v***_***d***_. According to ref.^19^, the radial dipole moment |*p*_*r*_| is calculated to be in the order of 10^−21^ C∙m for the dust particle having a similar size as our ice particles. Using this value, we can theoretically calculate the angular speed of the rotation by solving ${\overrightarrow{\tau }}_{E}+{\overrightarrow{\tau }}_{D}=0$ and it was found that *ω* is about few hundreds of rad/s, which is in good agreement with our observation.

**Figure 5:**
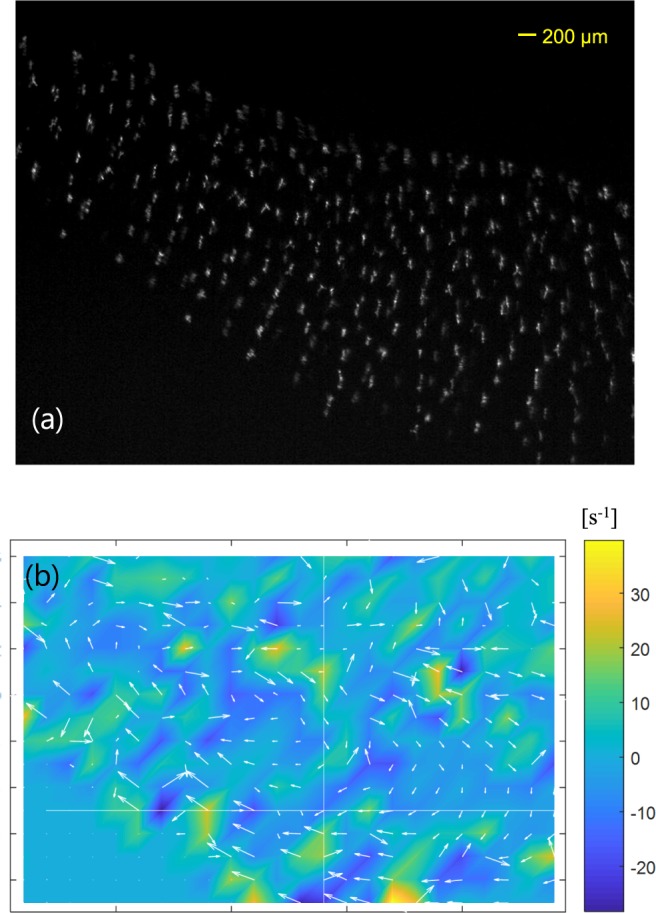
Vortex motion of nonspherical water-ice particles in a plasma. (**a**) Vortex motion of nonspherical particles. The camera recorded the upper-left corner of the plasma. The exposure time was 0.5 ms. While water-ice particles swirl, the alignment of nonspherical particles does not break up but the alignment angle slightly changes to be perpendicular to the cloud edge. In addition, nonspherical particles rotate about their alignment axis while they show vortex motion. (**b**) Velocity vectors (white arrows) obtained by an open source particle image velocimetry (PIV) analysis^28^. The length of vector corresponds to the speed of vortex flow. The color code represents the curl of the vortex velocity. Here, the cylindrical coordinate is used and so the positive means the direction out of the page.

Figure 5(a) is a still image of M3 (movie clip, available online) showing a vortex motion of nonspherical ice particles. The camera recorded the upper-left corner of the plasma. The camera exposure time was 0.5 ms and the frame rate was 300 fps. As shown in the figure (movie), nonspherical ice particles are rotating in the clockwise direction. It is not shown here but there is also a vortex flow in the upper-right corner and the direction of a vortex flow is in the counter-clockwise direction; vortex flows are axisymmetric.

It is found that the alignment of individual particles does not break up while ice particles show the vortex motion. The alignment orientation adjusts to be perpendicular to the edge line of the dust cloud when the particles pass the edge region of the dust cloud. Nonspherical particles swirl while rotating about their alignment axis. The rotation frequency is tens of Hz as seen in Fig. 4(a–d).

Figure 5(b) shows the velocity of the dust vortex flow and the curl of the flow velocity. In the calculation, we used an open source PIV code^29^. As shown in the movie the flow velocity is fast (>5 cm/s) near the top and bottom but relatively slow (2 cm/s) when the particles move upward and downward. The color code in the figure indicates the curl of vortex flow and the nominal value of the curl of vortex flow is in the order of 10 s^−1^.

The vortex motion is described by the cylindrical vorticity equations shown below^30^:

3 $${{\bf{u}}}_{{\boldsymbol{d}}}=(\nabla {\rm{\Psi }}\times \nabla \varphi )/2{\rm{\pi }}$$

4 $${r}^{2}\nabla \cdot (\nabla {\rm{\Psi }}/{r}^{2})=-\,2{\rm{\pi }}\chi $$

5 $$\frac{\partial }{\partial t}(\frac{\chi }{{r}^{2}})+{{\boldsymbol{u}}}_{{\boldsymbol{d}}}\cdot \nabla (\frac{\chi }{{r}^{2}})+{{\rm{\gamma }}}_{dn}(\frac{\chi }{{r}^{2}})=\frac{1}{r}\frac{{(\nabla \times {\bf{F}})}_{\varphi }}{{m}_{d}}+{\upsilon }_{d}\nabla \cdot (\frac{1}{{r}^{2}}\nabla \chi )$$

Here, **u**_**d**_ is the velocity of a dust fluid, Ψ is the fluid stream function, *χ* is the cylindrical vorticity defined as $\chi =r{(\nabla \times {{\bf{u}}}_{{\bf{d}}})}_{\varphi },\,{\gamma }_{dn}$ is the friction coefficient which is defined as *γ*_*dn*_ = 4π*r*_*d*_^2^*n*_*n*_*v*_*th*,*n*_*m*_*n*_/3*m*_*d*_,^28^ *ν*_*d*_ is the kinematic viscosity of a dust fluid, and **F** is the net force exerted on ice particles. We believe that the nonconservative ion drag force exerted on ice particles is responsible for the observed vortex flow^30–32^. To check the order of magnitude of the kinematic viscosity *ν*_*d*_, we compare two terms on the right-hand side of Eq. (5); the vorticity source term and the viscosity dissipation term (a steady-state solution with ignoring the friction term for the simplicity). The scale of the vortex flow shown in Fig. 5 is in the order of mm and thus the cylindrical vorticity χ is in the order of 10^−2^. The ion drag force exerted on dust particles is given by^33,34^ **F**_**id**_ = [*b*_*coll*_^2^ + *2b*_90_^2^Λ]π*n*_*i*_*m*_*i*_*v*_*i*_**u**_**i**_ where *b*_*coll*_ = *r*_*d*_[1 − (2*eV*_*d*_/*m*_*i*_*v*_*i*_^2^)]^1/2^, *b*_*90*_ = –*r*_*d*_(*eV*_*d*_/*m*_*i*_*v*_*i*_^2^), Λ = ln[(λ_s_^2^ + *b*_*90*_^2^)/(*b*_*coll*_^2^ + *b*_*90*_^2^)], *n*_*i*_ is the ion density, *m*_*i*_ is the ion mass, *u*_*i*_ is the ion flow velocity, *v*_*i*_ is the ion total speed, and *V*_*d*_ is the floating potential of the dust particles. The ion drag force is in the order of 10^−12^ N and the mass is in the order of 10^−11^ kg. Using these values, we can see that *ν*_*d*_ is in the order of 10^−4^ m^2^/s which is about 10 times larger than the value typically used in this field (see refs^35,36^). This might result from the fractal/nonspherical shape (or size) but further study is needed.

The detailed dynamics of nonspherical, fractal-like water-ice particles were observed and analyzed in a laboratory experiment running at an astrophysically relevant temperature. Several physical intuitions on the dynamics of nonspherical, fractal-like particles are obtained; (i) the mass of fractal particles is lighter than what they seem, (ii) elongated, fractal-like particles tend to align in the direction perpendicular to the force equilibrium line, (iii) nonspherical particles rotate about the alignment axis owing to the interaction between the dipole moment and the electric field, (iv) nonspherical particles show a vortex motion while maintaining their aligned structure if there is a nonconservative force acting on the particle, and (v) the kinematic viscosity of nonspherical, fractal particles seems larger than that of spherical particles.

## Electronic supplementary material

Supplementary Information

Behavior of particles after plasma turns off

Rotation of nonspherical particles

Vortex motion of nonspherical particles
