# Supplementary material for: Dynamics of nonspherical, fractal-like water-ice particles in a plasma environment
Source: Sci Rep. 2018 Oct 18;8:15405. doi: 10.1038/s41598-018-33854-5 (PMC6194125; doi:10.1038/s41598-018-33854-5)
Supplement: Supplementary file 1 — Supplementary Information [file 41598_2018_33854_MOESM1_ESM.docx]

**Supplementary Information: Dynamics of nonspherical, fractal-like water-ice particles in a plasma environment**

Kil-Byoung Chai^1^

Korea Atomic Energy Research Institute, Daejeon 34067, South Korea. Correspondence and requests for materials should be addressed to K.-B. C. (email: kbchai@kaeri.re.kr)

^*^ kbchai@kaeri.re.kr

**M1**. **Behavior of particles after plasma turns off**

Animated file shows that water-ice particles formed in our laboratory experiment move upward after the plasma is turned off. This implies that the upward thermophoretic force overwhelms the downward gravitational force due the temperature difference between the top and bottom electrodes. The recorded frame rate was 300 fps and the movie is displaying at 10 fps. The exposure time was 0.5 ms.

**M2**. **Rotation of nonspherical particles**

Movie file shows that nonspherical particles rotate about their alignment axis. The rotation frequency typically ranges 20-50 Hz. The recorded frame rate was 300 fps and the movie is now displaying at 10 fps. The exposure time was 0.5 ms.

**M3**. **Vortex motion of nonspherical particles**

Movie clip describes vortex motion of nonspherical particles. Under the nonconservative drag force (here, the ion drag force) they swirl while maintaining their aligned structure and rotation about their alignment axis.
